# Editorial: Seizure Forecasting and Detection: Computational Models, Machine Learning, and Translation Into Devices

**DOI:** 10.3389/fneur.2022.874070

**Published:** 2022-03-16

**Authors:** Sharon Chiang, Maxime O. Baud, Gregory A. Worrell, Vikram R. Rao

**Affiliations:** ^1^Department of Neurology and Weill Institute for Neurosciences, University of California, San Francisco, San Francisco, CA, United States; ^2^Department of Neurology, Sleep-Wake-Epilepsy Center and Center for Experimental Neurology, Inselspital Bern, University Hospital, University of Bern, Bern, Switzerland; ^3^Wyss Center for Bio- and Neuro-Technology, Geneva, Switzerland; ^4^Department of Neurology, Mayo Clinic, Rochester, MN, United States

**Keywords:** epilepsy, seizure detection, seizure prediction, seizure forecasting, computational models, machine learning, artificial intelligence

For the 50 million people with epilepsy worldwide, seizures are seemingly unpredictable events that can be associated with significant morbidity and mortality. Reliable methods to identify and anticipate seizures could enable powerful new therapeutic strategies. Owing to the paroxysmal nature of seizures and the challenges associated with long-term monitoring of brain activity, however, such methods have been elusive—until now. Recent advances in computational methods and device technology have reshaped the epilepsy landscape and made accurate detection and forecasting of seizures a reality. This Research Topic was launched with the aims of highlighting the latest of these advances and portraying the current state of seizure detection and forecasting through the viewpoints of patients and researchers. The 18 articles in this special issue comprise three Perspectives, three Reviews, and 12 Original Research articles.

This collection begins with a first-person account from Moss et al. of one family's experience with drug-resistant epilepsy, including their appraisal of current seizure detection devices; challenges encountered related to privacy, stigma, and comfort; and the need for greater collaboration between patients and researchers, Grzeskowiak and Dumanis survey a large sample of caregivers and adults living with epilepsy to assess directly their perspectives on seizure forecasting, including the optimal forecast horizon, the types of information that would be most useful for day-to-day planning, and the potential risks of forecasting tools. Hubbard et al. and Brinkmann et al. review the current status, technical challenges, and performance characteristics of contemporary seizure detection devices. Both groups highlight the critical need for research on several fronts: robustly designed clinical validation studies; algorithms to detect seizure types in addition to convulsive seizures (CS); better incorporation of meaningful clinical outcomes—such as morbidity, mortality, and quality of life—into algorithm evaluation; and more emphasis by developers on patient-centered considerations prior to beta-testing of new algorithms.

Several research groups whose work is included in this issue have already made substantial progress toward these goals. Glaba et al. and Kjaer et al. describe promising new algorithms that leverage physiological measurements to detect seizure types other than CS, including absence seizures, using electroencephalography (EEG), and focal seizures, using subcutaneous EEG, accelerometry, and electromyography. Although collection of these types of physiological data is essential for seizure detection, patient privacy and unobtrusiveness of devices are also paramount considerations in algorithm and technology development. In this context, Manzouri et al. quantify and compare the relative energy efficiency and performance of several common machine learning methods, including random forests, recurrent (e.g., long short-term memory), and convolutional neural networks. Hyperdimensional computing, reviewed in an accessible tutorial by Schindler and Rahimi, is proposed as a powerful approach to develop energy-efficient seizure detection algorithms. Similar considerations of efficiency and performance are relevant for devices being developed for seizure detection. Frankel et al. report on a prospective feasibility study of Epilog by Epitel, Inc., a wearable 10-channel EEG system for long-term recordings. The authors recruited expert EEG readers and evaluated their ability to detect seizures using Epilog with or without a clinical decision support system. Onorati et al. conducted the first prospective multi-center study of a multimodal CS detection system, based on a wrist-worn device combining accelerometers and electrodermal activity sensors, and they report excellent sensitivity and a low false alarm rate.

Seizure detection algorithms also promise improvements to epilepsy diagnostic evaluations. As discussed by Parasuram et al. computational methods that accommodate spatial and temporal features of EEG may be more useful for identifying the ictal onset zone compared to methods relying on temporal features alone. Li et al. provide promising evidence that deep neural networks can identify seizures that are occult on scalp EEG but visible in intracranial EEG. These neural networks can also achieve high prediction accuracy for other tasks, including ictal onset zone lateralization and discrimination between ictal, preictal, and interictal periods.

Seizure detection and seizure forecasting represent complementary strategies for mitigating morbidity in epilepsy. Whereas, seizure detection provides a means to alert caregivers to seizures when they occur, seizure forecasting aims at reducing uncertainty by quantifying the likelihood of seizures in the future (1–3). The niche endeavor of forecasting seizures already has a decades-long history (4, 5), but has only recently gained traction among clinicians and patients, as the recent development of devices and algorithms makes it a plausible near-reality. Brinkmann et al. review variables that can be incorporated into seizure forecasting algorithms, including cycles of brain activity that help determine seizure risk (6, 7). Karoly et al. delve into these seizure cycles and debunk several common misconceptions related to seizure forecasting. For example, the authors clarify that cycles in epilepsy cannot be explained solely by behavioral patterns, medications, or catamenial effects, and they argue that long timescale rhythms in epilepsy may reflect systemic physiological processes that are cyclical and that manifest across a range of human diseases (Karoly et al.). This view agrees with the identification of multidien rhythms of epileptic brain activity as free-running, and thus likely endogenous in nature (8, 9).

The feasibility of seizure prediction on a population scale hinges on the development of minimally- and non-invasive devices. In a study of 11 patients with wearable devices, Stirling, Grayden et al. demonstrate that seizure likelihood can be forecasted with better than chance-level accuracy by using algorithms that incorporate data on heart rate, sleep, and step counts. Stirling, Maturana et al. show that seizure prediction is also possible with subcutaneous EEG (10) by using a state-based approach involving a two-step combination of logistic regression and random forests. Despite growing interest in potential applications of subcutaneous EEG (11), scalp EEG remains far more widely available, so Truong et al. use scalp EEG data and a Bayesian convolutional neural network to predict seizures in three patients. Attia et al. describe the design and architecture of the Mayo Epilepsy Personal Assistant Device, a cloud-based mobile platform integrated with an implanted intracranial neurostimulation device that is being used in an ongoing trial of neurostimulation in ten patients with bilateral mesial temporal lobe epilepsy. This special issue concludes with a cautionary note from Bosl et al. who draw upon experience from seizure detection efforts to highlight both the promise of seizure forecasting and the need for tempered optimism in a burgeoning field.

The 18 articles featured in this issue provides a status update and insight into representative lessons learned thus far in the years since the goals set by colleagues Mormann et al. (4) and Freestone et al. (5). In summary, we offer the reader the following insights on the current status of seizure detection and forecasting and several important priorities that emerge from this special issue:

1. **Emphasis on multimodal techniques:** There is no single perfect data stream; seizure detection and forecasting approaches will benefit from increased focus on statistical methods that accommodate multimodal data.
2. **Focus on understanding patient priorities and patient-researcher partnerships:** Patient perspectives are crucial to aid understanding of essential algorithm and device design aspects early in the development cycle, and to ensure that patient priorities maintain primacy in algorithm/device development (12).
3. **Interpretability and explainability in machine learning:** Machine learning methods may identify insights that humans cannot. Interpretability and explainability of predictions are active areas of research. We anticipate that the coming years will see translation of emerging computational techniques to enhance explainability into the area of seizure detection/forecasting.
4. **Expansion of seizure detection to other seizure types:** Current seizure detection methods achieve excellent performance using EEG. Detection algorithms with non-EEG signals have attained high sensitivity and specificity for certain types of seizures (convulsions), but remain limited for detection of other seizure types. Communicating the nuanced limitations of these methods to the patient community is just as critical as research on algorithms to detect non-convulsive seizure types. Developing technology to quantitatively measure patient behavior and consciousness may help advance seizure classification.
5. **Need for increased studies evaluating reproducibility/generalizability:** Studies are emerging which seek to evaluate the reproducibility and generalizability of seizure detection/forecasting algorithms, and additional development will be needed to translate these algorithms into real-world settings. Expanding the sensing, data storage, and streaming capabilities of the next generation of devices will facilitate seizure detection/forecasting algorithm development.
6. **Computational efficiency vs. performance:** Given the importance to patients of non-stigmatizing devices, designing computationally efficient algorithms, balancing computational efficiency vs. performance, and developing minimally-invasive devices are research priorities.
7. **Individualized algorithm development:** Patients are heterogeneous in their seizure patterns and semiologies, as well as in the salient variables and non-linear functions relating these variables. A patient whose seizures may not be detected/forecasted by one algorithm and/or set of variables may be well-predicted by another. It is likely that multiple methods will need to be combined and tailored based on patient-specific factors.
8. **No prediction or detection method is perfect:** Seizure forecasts reduce uncertainty but do not eliminate it. The hope is that quantified uncertainty will translate into improved quality of life for people with epilepsy, but, although reasonable, this leap of faith will require direct testing in the clinical setting.

## Author Contributions

SC, MOB, GAW, and VRR: manuscript concept, design, and final revision. All authors contributed to the article and approved the submitted version.

## Funding

SC was supported by the National Institute of Neurological Disorders and Stroke, National Institutes of Health (5R25NS070680-12). GAW was supported by the National Institutes of Health UH2/UH3 NS95495.

## Conflict of Interest

MOB reports personal fees and grants from the Wyss Center for neurotechnology in Geneva, and has a patent application pending under the Patent Cooperation Treaty (62665486). MOB holds shares with Epios, Ltd., a medical device company based in Geneva. GAW declares intellectual property disclosures related to brain state classification algorithms licensed to Cadence Neuroscience Inc. VRR has served as a consultant for NeuroPace, Inc., manufacturer of the RNS System. The remaining author declares that the research was conducted in the absence of any commercial or financial relationships that could be construed as a potential conflict of interest.

## Publisher's Note

All claims expressed in this article are solely those of the authors and do not necessarily represent those of their affiliated organizations, or those of the publisher, the editors and the reviewers. Any product that may be evaluated in this article, or claim that may be made by its manufacturer, is not guaranteed or endorsed by the publisher.

## References

[1] Proix T Truccolo W Leguia MG Tcheng TK King-Stephens D Rao VR. Forecasting seizure risk in adults with focal epilepsy: a development and validation study. Lancet Neurol. (2021) 20:127–35. 10.1016/S1474-4422(20)30396-3 33341149 PMC7968722

[2] Karoly PJ Cook MJ Maturana M Nurse ES Payne D Brinkmann BH. Forecasting seizure likelihood with wearable technology. Epilepsia. (2020) 61:776–86. 10.1111/epi.16485 32219856

[3] Kuhlmann L Karoly P Freestone DR Brinkmann BH Temko A Barachant A. Epilepsyecosystem.org: crowd-sourcing reproducible seizure prediction with long-term human intracranial EEG. Brain. (2018) 141:2619–30. 10.1093/brain/awy210 30101347 PMC6136083

[4] Mormann F Andrzejak GR Elger EC Lehnertz K. Seizure prediction: the long and winding road. Brain. (2007) 130(Pt 2):314–33. 10.1093/brain/awl241 17008335

[5] Freestone DR Karoly PJ Cook MJ. A forward-looking review of seizure prediction. Curr Opin Neurol. (2017) 30:167–73. 10.1097/WCO.0000000000000429 28118302

[6] Baud MO Kleen JK Mirro EA Andrechak JC King-Stephens D Chang EF. Multi-day rhythms modulate seizure risk in epilepsy. Nat Commun. (2018) 9:88. 10.1038/s41467-017-02577-y 29311566 PMC5758806

[7] Karoly PJ Goldenholz DM Freestone DR Moss RE Grayden DB Theodore WH. Circadian and circaseptan rhythms in human epilepsy: a retrospective cohort study. Lancet Neurol. (2018) 17:977–85. 10.1016/S1474-4422(18)30274-6 30219655

[8] Rao VR Leguia GM Tcheng TK Baud MO. Cues for seizure timing. Epilepsia. (2021) 62(Suppl. 1):S15–31. 10.1111/epi.16611 32738157

[9] Baud MO Ghestem A Benoliel JJ Becker C Bernard C. Endogenous multidien rhythm of epilepsy in rats. Exp Neurol. (2019) 315:82–7. 10.1016/j.expneurol.2019.02.006 30776337

[10] Duun-Henriksen J Baud M Richardson MP Cook M Kouvas G Heasman JM. A new era in electroencephalographic monitoring? Subscalp devices for ultra-long-term recordings. Epilepsia. (2020) 61:1805–17. 10.1111/epi.16630 32852091

[11] Pathmanathan J Kjaer TW Cole AJ Delanty N Surges R Duun-Henriksen J. Expert perspective: who may benefit most from the new ultra long-term subcutaneous EEG monitoring? Front Neurol. (2021) 12:817733. 10.3389/fneur.2021.817733 35126304 PMC8810530

[12] Chiang S Picard RW Chiong W Moss R Worrell GA Rao VR. Guidelines for conducting ethical artificial intelligence research in neurology: a systematic approach for clinicians and researchers. Neurology. (2021) 97:632–40. 10.1212/WNL.0000000000012570 34315785 PMC8480407

